# Potentially Traumatic Events, Posttraumatic Stress Disorder, and Depression among Adults in Puerto Rico

**DOI:** 10.3389/fpsyg.2016.00469

**Published:** 2016-03-31

**Authors:** Cassie Overstreet, Erin C. Berenz, Christina Sheerin, Ananda B. Amstadter, Glorisa Canino, Judy Silberg

**Affiliations:** ^1^Virginia Institute for Psychiatric and Behavioral Genetics, Virginia Commonwealth University, RichmondVA, USA; ^2^Developmental Pediatrics, University of Virginia, CharlottesvilleVA, USA; ^3^Behavioral Sciences Research Institute, University of Puerto Rico, San JuanPR, USA

**Keywords:** posttraumatic stress disorder, trauma, depression, Puerto Rico, resilience

## Abstract

The aims of the current study were to examine the prevalence of potentially traumatic events (PTEs), posttraumatic stress disorder (PTSD; data available in males only), and depressive symptoms in a Puerto Rican sample of 678 adult caretakers (50% female) of twins participating in the Puerto Rican Infant Twin Study. The World Health Organization Composite International Diagnostic Interview version 3.0 (CIDI 3.0) was utilized to assess rates of PTEs, PTSD, and depression among male participants while an abbreviated version of the CIDI 3.0 and the Mood and Feelings Questionnaire were administered to females to assess PTEs and depressive symptoms. Significantly more males than females reported exposure to a PTE (76.6% vs. 44.2%, χ^2^ = 64.44, *p* < 0.001). In males, endorsement of multiple PTEs was associated with increased level of PTSD symptomatology (β = 0.33, *p* < 0.001). With regard to depression, a similar dose-response relationship was found in both males and females, with depressive symptoms increasing as number of PTEs increased (βs = 0.15, 0.16, *ps* < 0.05). Exposure to an attack with a weapon was significantly associated with increased depression symptoms in both males and females (βs = 0.24, 0.20, *ps* < 0.01, respectively). These findings highlight the need for identification of putative risk and resilience factors among PTE-exposed individuals in Puerto Rico.

## Introduction

Exposure to potentially traumatic events (PTEs; i.e., exposure to actual or threatened death, serious injury, or sexual violence; American Psychiatric Association, 2013) is associated with increased rates of psychopathology in numerous regions around the world (Pine et al., 2005; Stein et al., 2010) and significantly contribute to risk for posttraumatic stress disorder (PTSD) and other stress-related psychiatric disorders (Breslau, 1998). Although the association between PTEs and psychopathology has been well established in high-income Western regions (e.g., Kessler et al., 1995; Perkonigg et al., 2000), fewer studies have examined a wide range of PTEs in lower to middle income regions. Following, the purpose of the present study is to determine the prevalence of interpersonal and accidental PTEs in a large sample of adults in Puerto Rico. Additional aims included examining the relationship between both exposure to multiple types of trauma and the relationship between trauma type and psychopathology.

Although PTE exposure is generally related to elevated risk of psychopathology across cultures, the rates and types of PTEs experienced vary significantly by geography and culture (Breslau, 1998; Marques et al., 2011). Debate exists regarding whether post-trauma psychopathology, specifically PTSD, is a Western “culture bound syndrome” and whether it can be adequately applied to other cultures (Summerfield, 1999). However, discrepancies in prevalence rates may also be due to methodological issues (e.g., how trauma is defined, lack of assessment of culturally relevant responses; Hinton and Lewis-Fernández, 2011). Further, the relationship between PTE exposure and psychopathology across diverse regions lack comprehensive review (Kira, 2010). This gap is underscored by unsuccessful implementation of mental health initiatives, based on work from the continental US, for trauma-exposed individuals in other parts of the world (Walker and Walter, 2000).

The extant literature on variability in outcomes following PTE exposure suggests that event characteristics are related to risk of psychopathology (Breslau et al., 1999). This includes trauma type, with interpersonal trauma (e.g., sexual assault, physical assault) conferring the greatest level of risk for development of PTSD (Breslau, 1998; Breslau et al., 1998; Frans et al., 2005) as well as greater trauma load (i.e., number of lifetime traumatic events; Neuner et al., 2004; Howgego et al., 2005). This suggests a cumulative effect, whereby the experience of multiple PTEs contributes to PTSD and depressive symptoms that otherwise would not occur in the instance of a single event (Sapolsky, 1998; Resnick et al., 2003).

Social and community-level factors also appear to impact the experience of PTEs and associated psychopathology. Previous research has demonstrated that individuals within the lower socio-economic strata experience elevated risk for stressful life events and multiple-risk exposures (i.e., the experience of more than one significant stressor at a single time, such as trauma, teenage pregnancy, and divorce; Evans and Kim, 2010). In turn, limited access to social and economic resources, such as economic hardship (Fryers et al., 2003; Skapinakis et al., 2006; Ahnquist and Wamala, 2011; McLaughlin et al., 2011) and limited social support (Kessler et al., 1985; Khantzian, 1985; Maulik et al., 2010) may be more prevalent in low-to middle-income regions and related to negative mental health outcomes (Gerra et al., 2000).

Puerto Rico has traditionally been considered an area of low- to middle-income level with 45.5% of the population living below the poverty line (Lupien et al., 2009). Due to the putative relationship between economic hardship and psychopathology, studies have examined the prevalence and correlates of psychiatric conditions in Puerto Rico. Findings have been mixed, with some identifying higher rates within a Puerto Rican sample relative to U.S.-based populations (Shrout et al., 1992) while others have demonstrated similar rates between island Puerto Rico and mainland U.S. (Canino et al., 1987, 2004). Although research exists regarding the prevalence of post trauma psychopathology in Puerto Rico (Canino et al., 1990; Felix et al., 2011), limited work has addressed the prevalence of different PTE types, the relationship between number of PTE types experienced and mental health outcomes, and the relationship between PTE types and mental health outcomes. The limited research on PTE exposure in Puerto Rico predominately pertains to mental health outcomes following natural disasters (Guarnaccia et al., 1993; Koob and Le Moal, 2001; Felix et al., 2011; Rivera, 2012) and childhood trauma (Kirschbaum et al., 1993; Francia-Martinez et al., 2003) with less work focused on cumulative exposure across multiple types of PTEs, a known risk factor (Mollica et al., 1998; Kaysen et al., 2010).

The dearth of research on PTE characteristics (i.e., load, type) and post-trauma psychiatric outcomes among Puerto Ricans highlights this understudied area within the literature and warrants further investigation of these variables as they relate to this population and region. Thus, the aim of this study was to assess the rates of PTE types and the relationships between PTE characteristics (i.e., load, type) and psychiatric outcomes (i.e., PTSD in males and depressive symptoms in both sexes) among adult caregivers in Puerto Rico. Given that the present study was completed as a part of larger project examining health among a population of children in Puerto Rico, caretaker assessment was necessarily abbreviated to reduce participant burden, particularly in women as mothers also had to respond to items pertaining to their children. We hypothesized that a high rate of PTEs would be endorsed and that individuals possessing a history of lifetime PTE exposure would report significantly more symptoms of PTSD (in males only) and depression (in males and females) than individuals without a PTE history. Furthermore, we expected higher PTE load and PTEs considered more interpersonal in nature would be associated with increased PTSD and depressive symptoms.

## Materials and Methods

### Participants

Participants were 678 adult caregivers (50% female; *M_age_* = 28.2, *SD* = 6.4) enrolled in the Puerto Rican Infant Twin Study (PRINTS). Families were identified via the Puerto Rico Neo-natal Twin Registry, established with aid from the Puerto Rican Department of Health. Of the 399 eligible families invited to participate in the study, 339 families consented.

### Procedure

The study obtained parental data from 2006 to 2007 with each parent separately interviewed within the first year of their child’s life concerning demographic information, lifetime PTEs, and psychopathology. Mothers provided data on the children’s psychological functioning as well; therefore, to reduce participant burden, mothers completed an abbreviated self-assessment (detailed below). Interviews were conducted and taped in each family’s place of residence, with different interviewers assessing each parent separately and blinded to the results of the other assessment. Fifteen percent of interviews were spot-checked for quality control. The Institutional Review Board of the University of Puerto Rico and Virginia Commonwealth University approved the study, and informed consent was obtained from all participants. Lifetime PTEs were assessed via a revised version of the Spanish translation of the World Health Organization Composite International Diagnostic Interview version 3.0 PTSD module. Past year PTSD and major depressive episode symptoms were assessed with the same measure for males. Symptoms of depression in females were assessed via the Short Mood and Feelings Questionnaire.

### Measures

World Health Organization Composite International Diagnostic Interview version 3.0 (CIDI 3.0; Kessler and Ustun, 2004) is a standardized diagnostic interview that allows for evaluation of current and lifetime psychopathology based on the *Diagnostic and Statistical Manual of Mental Disorders Fourth Edition* (DSM-IV-TR; American Psychiatric Association, 2000). For use in this specific population, the instrument was adapted and translated into Spanish using a cultural adaptation model stressing cross-cultural equivalence across five dimensions (Alegria et al., 2004). The CIDI 3.0 questions concerning exposure to lifetime PTEs were altered from 26 items regarding specific PTEs to 10 items that combined similar items (e.g., a single item was used for physical assault without a weapon, combining the three separate items for family member, romantic partner, or anyone else). Lifetime history of PTEs assessed included natural disaster, serious accident, attack with a weapon, attack without a weapon, military combat, sexual assault, and other situation involving serious injury, other situation involving fear of injury or death, viewing serious injury or violent death, and any other extraordinarily stressful event. PTSD symptoms in the past year were assessed among participants endorsing at least one PTE; participants could respond to the PTSD symptoms in reference to any of the PTEs they experienced. The full CIDI 3.0 was administered to fathers and was used to index total number of PTEs, specific PTE categories, past year PTSD symptoms, and assess lifetime major depressive episode (MDE) symptoms. Mothers responded to the revised CIDI 3.0 PTE items only.

The Short Mood and Feelings Questionnaire (SMFQ; Angold et al., 1995) is a 13-item self-report questionnaire frequently used in epidemiologic studies derived from the 34-item Mood and Feelings questionnaire and designed to assess symptoms of depression in the past 3 months (MFQ; Li et al., 2012). The SMFQ has good internal reliability (α = 0.90; Herrman et al., 2011; α = 0.78 within the present sample). Responses are made on a three-point scale (0 = “not true,” 1 = “sometimes,” 2 = “true”). A continuous sum score was calculated with a response of “true” to any item equaling 1 point and higher scores indicating greater symptoms (Lange et al., 2011). The translation to Spanish and adaptation for use among Latino participants used a five-step model for obtaining cross-cultural equivalence (Matías-Carrelo et al., 2003).

### Data Analytic Plan

Descriptive analyses were conducted to examine rates of lifetime PTEs among males and females. Univariate tests (Chi-square analyses) were conducted to compare PTEs between males and females. Hierarchical linear regressions were conducted to examine the relationship between continuous number of PTE types endorsed and both MDE symptoms and PTSD symptoms in men and past 3-month depressive symptoms in women per the MFQ. Additional regression analyses were also conducted to examine PTEs reported (i.e., serious accident, natural disaster, sexual assault, injury, attack with a weapon, attack without a weapon, witnessed violence) as predictors. Covariates of age and education level were included in all regression analyses. All analyses were conducted in SPSS, version 21 and an alpha level of 0.05 was used for all analyses.

## Results

### Sample Characteristics

Within the sample 51.8% of men and 40.1% of women possessed at least a high school education. The mean age of men was 29.66 (*SD* = 6.9 years) and 26.79 (*SD* = 5.94) for women. Additional baseline characteristics of parents participating in the PRINTS can be located in Lange et al. (2011).

### Lifetime PTE Exposure in Men

Potentially traumatic event exposure was common in men, with 76.6% (*n* = 216) endorsing at least one lifetime PTE (**Table 1**), of which natural disaster was the most common (53.2%, *n* = 150). The average number of PTE types endorsed was 2.13 (*SD* = 2.07). When specific PTEs were clustered, 41.5% (*n* = 117) endorsed an interpersonal PTE, 67.7% (*n* = 191) reported an accidental PTE, and 29.1% (*n* = 82) endorsed an other PTE.

**Table 1:** Prevalence of lifetime potentially traumatic events among Puerto Rican males and females.

	Males (*n* = 338)	Females (*n* = 340)	χ^2^	*p*
Any PTE endorsed	76.6%	44.2%	64.44	<0.001
PTE types				
Serious accident	38.3%	11.9%	56.11	<0.001
Natural disaster	53.2%	25.3%	48.57	<0.001
Sexual assault	1.1%	5.4%	8.75	0.003
Attack (with weapon)	21.3%	6.9%	26.61	<0.001
Attack (without weapon)	17.4%	5.4%	21.34	<0.001
Military combat	2.5%	0.3%	5.21	0.022
Injury	9.6%	3.8%	7.92	0.005
Witnessed violence	31.2%	8.3%	49.97	<0.001
Other (feared injury/death)	18.4%	8.3%	13.26	<0.001
Other (stressful event)	19.9%	13.5%	4.40	0.036


*χ^2^ = chi-square.*

### Lifetime PTE Exposure in Women

Potentially traumatic event exposure was also relatively common in women, with 44.2% (*n* = 138) reporting exposure to at least one lifetime PTE (**Table 1**), with natural disaster again the most common type reported (25.3%, *n* = 79). When specific PTEs were clustered, 17.1% of all respondents (*n* = 53) endorsed an interpersonal event as potentially traumatic, 33% (*n* = 103) reported an accidental PTE, and 17.9% (*n* = 56) endorsed an other PTE. The average number of PTE types endorsed was 0.89 (*SD* = 1.41). Overall, women were less likely to experience a PTE in their lifetime (χ^2^ = 64.44, *p* < 0.001) and endorsed fewer exposures across the various types with the exception of sexual assault (χ^2^ = 8.75, *p* < 0.01).

### Lifetime PTEs, PTSD Symptoms, and Major Depressive Episode Symptoms in Men

Of those endorsing a lifetime PTE, 6.7% (*n* = 14) met criteria for PTSD. Among males reporting at least one traumatic experience, number of lifetime PTE types was significantly associated with higher levels of past year PTSD symptoms, after controlling for age and education level (β = 0.33, *p* < 0.001). Furthermore, when examining the relationship between PTE type and PTSD symptoms, report of an injury was significantly associated with PTSD symptoms (β = 0.16, *p* = 0.03). Per the CIDI Depression module, 12.1% (*n* = 34) met criteria for lifetime MDE and 12.1% (*n* = 4) for both lifetime MDE and PTSD. 73.5% (*n* = 25) of those meeting criteria for lifetime MDE endorsed experiencing at least one PTE in their lifetime (χ^2^ = 0.20, *p* = 0.65). Furthermore, 67.6% (*n* = 23) endorsing an interpersonal PTE (χ^2^ = 10.90, *p* = 0.001), 58.8% (*n* = 20) endorsing an accidental PTE (χ^2^ = 1.40, *p* = 0.24), and 47.1% (*n* = 16) endorsing an event categorized as other (χ^2^ = 6.06, *p* = 0.01) met criteria for lifetime MDE. Number of PTE types endorsed was significantly associated with symptoms of lifetime MDE (β = 0.15, *p* = 0.01; **Table 2**). In the hierarchical regression using PTE type, only attack with a weapon was significantly associated with increased depressive symptoms (β = 0.24, *p* < 0.001; **Table 3**).

**Table 2:** Number of lifetime potentially traumatic events predicting lifetime major depressive episode symptoms in males (*n* = 282).

	*R*^2^	*t*	β	*p*
Model 1	0.01			
Age		0.41	0.03	0.68
Education		-0.002	0.001	0.99
Model 2	0.02			
Age		0.45	0.03	0.66
Education		-0.29	-0.02	0.78
PTE load		2.48	0.15	0.01


**R*^2^ = Coefficient of determination.*

**Table 3:** Potentially traumatic event type predicting lifetime major depressive episode symptoms in males (*n* = 282).

	*R^2^*	*t*	β	*p*
Model 1	0.001			
Age		0.41	0.03	0.68
Education		-0.002	0.001	0.99
Model 2	0.09			
Age		0.76	0.05	0.50
Education		-0.41	-0.03	0.68
Serious accident		-1.27	-0.08	0.21
Natural disaster		-1.86	-0.11	0.06
Sexual assault		0.20	0.01	0.84
Attack (with weapon)		3.33	0.24	0.001
Attack (without weapon)		0.64	0.05	0.52
Military combat		-0.64	-0.04	0.53
Injury		0.03	0.002	0.98
Witnessed violence		1.07	0.07	0.29


**R*^2^ = Coefficient of determination.*

### Lifetime PTEs and Past 3-Month Depression Symptoms in Women

Women with lifetime PTE history compared to those without did not differ on MFQ total scores [*t*(309) = 9.38, *p* = 0.22]. Greater lifetime PTEs was associated with higher past 3-month depressive symptoms, after controlling for the covariates of age and education (β = 0.16, *p* < 0.01) (**Table 4**). The regression examining the relationship between PTE type and depressive symptoms demonstrated that exposure to an attack with a weapon was associated with higher past 3-month depressive symptoms (β = 0.20, *p* < 0.01; **Table 5**).

**Table 4:** Number of lifetime potentially traumatic events predicting past 3-month depressive symptoms in females (*n* = 311).

	*R*^2^	*t*	β	*p*
Model 1	0.05			
Age		-0.74	-0.05	0.46
Education		-3.20	-0.20	<0.01
Model 2	0.07			
Age		-0.85	-0.05	0.40
Education		-3.38	-0.21	<0.01
PTE Load		2.81	0.16	<0.01


**R*^2^ = Coefficient of determination.*

**Table 5:** Potentially traumatic event type predicting lifetime major depressive episode symptoms in females (*n* = 311).

	*R*^2^	*t*	β	*p*
Model 1	0.05			
Age		-0.74	-0.05	0.46
Education		-3.20	-0.20	<0.01
Model 2	0.10			
Age		-0.99	-0.06	0.33
Education		-3.25	-0.20	0.001
Serious accident		-0.20	-0.01	0.84
Natural disaster		0.34	0.02	0.74
Sexual assault		-0.86	-0.05	0.39
Attack (with weapon)		2.66	0.20	<0.01
Attack (without weapon)		0.71	0.05	0.48
Military combat		0.62	0.04	0.53
Injury		0.44	0.03	0.66
Witnessed violence		-0.70	-0.04	0.49


## Discussion

This study extends previous research regarding the prevalence rates of PTEs and associated psychiatric symptoms in an understudied population. A significant proportion of the Puerto Rican sample endorsed exposure to at least one lifetime PTE (76.6% of men and 44.2% of women). Although this sample is not epidemiologic in nature, preventing direct comparisons to mainland US epidemiologic studies that speak to statistical significance, to put our results in context, in epidemiologic studies of the mainland US approximately 60.7% of males and 51.2% of females report PTE exposure (Kessler et al., 1995). Our prevalence is more comparable when examined in relation to rates in male Latinos residing in mainland US (79.1%; Alegria et al., 2013). Similar to mainland US studies (Tolin and Foa, 2006), males were more likely to have a PTE history in our study, although some PTEs (e.g., adult sexual assault) were more prevalent in females, suggesting the need for sex-specific research on risk and protective factors for PTE exposure.

Among males, the low prevalence (6.7%) of past year PTSD may be indicative of culturally bound protective factors following PTE exposure unique to this population (e.g., methods of coping, means of social support; Canino et al., 1987). Additional research is necessary to better understand the factors that may confer protection within this particular population. However, the lower rates in this sample may also indicate that the present sample is not representative of the Puerto Rican population as a whole (Canino et al., 1987). Other factors may have also influenced the present findings, including the possibility that mental health may be less openly discussed (Rivera-Segarra et al., 2014) and potential differences in definitions of trauma across cultures (Wilson and Keane, 2004). These potential explanations for differential rates require further attention in order to understand the ways in which assessment methods and resilience factors may influence post-trauma response patterns. These concerns are not confined to research regarding post-trauma psychopathology in Puerto Rico but all cross-cultural research (Kessler and Bromet, 2013; Steel et al., 2014).

The results also suggest a relationship between number of lifetime PTEs and past year PTSD in males and number and type of PTE on depressive symptoms in both females and males. The present results suggest a dose response effect, whereby psychiatric symptoms significantly increased as amount of lifetime PTEs rise in both males and females, in line with findings of the cumulative effect of PTEs increasing psychiatric symptoms (Mollica et al., 1998; Kaysen et al., 2010; Shih et al., 2010) that would otherwise not exist or present to a lesser extent following a single event. The positive and specific association of PTEs considered more interpersonal in nature (i.e., attack with a weapon) on increased depression symptoms in both males and females aligns with established evidence in western populations of the potency of this trauma type on negative mental health outcomes, particularly depression (Cerdá et al., 2012). Findings also suggest that this association may extend to males (who area less studied) and across cultural boundaries.

The contribution of these findings to an understudied area should nonetheless be considered in the context of existing limitations. First, emotional reactions to (i.e., feelings of fear, helplessness, or horror) and perceived severity and timing of the individual PTEs were not assessed, resulting in uncertainty regarding the impact of any one event in particular. Second, the abbreviated version of the assessment battery for females resulted in less data available on psychiatric outcomes for this subset of the sample. Future studies utilizing more comprehensive assessment batteries for men and women alike will facilitate comparison by sex and culture. Finally, the current study compared observed rates of lifetime PTEs and associated symptoms with those obtained in mainland US samples; however, the current sampling approach (i.e., parents of twins in Puerto Rico) may limit generalizability to other samples, including those within Puerto Rico. Future epidemiologic studies in Puerto Rico would be particularly useful.

Despite its limitations, the present study provides valuable information regarding the prevalence of lifetime PTEs psychiatric outcomes in a Puerto Rican sample. The relatively low level of psychopathology in the sample compared to mainland US prevalence rates may be indicative of culturally bound factors associated with resilience (e.g., social networks in Puerto Rico; Canino et al., 1987), warranting additional investigation. Additionally, present findings highlight the importance of examining both number and type of PTEs in trauma research. More thorough investigation of the factors that may influence the relationship between PTEs and psychopathology in Puerto Rico is necessary to develop a better understanding of how each factor contributes and to what degree each can inform prevention and intervention programming.

## Author Contributions

CO, EB, CS, GC, AA, and JS each made substantial contributions to the conception or design of the work, or the acquisition, analysis, or interpretation of data for the work, drafting the work or revising it critically for important intellectual content, final approval of the version to be published, and agreed to be accountable for all aspects of the work in ensuring that questions related to the accuracy or integrity of any part of the work are appropriately investigated and resolved.

## Conflict of Interest Statement

The authors declare that the research was conducted in the absence of any commercial or financial relationships that could be construed as a potential conflict of interest.
